# 75. Relapsing polychondritis with tracheal involvement: responsive to induction cyclophosphamide therapy

**DOI:** 10.1093/rap/rky034.038

**Published:** 2018-09-20

**Authors:** Mohammad Solaiman, Olabambo Ogunbambi

**Affiliations:** Rheumatology, Hull Royal Infirmary, Hull, UNITED KINGDOM

**Introduction:** We report the case of a patient with relapsing polychondritis who presented with significant tracheal involvement (laryngeal stridor, exertional dyspnoea) partially responsive to initial induction therapy with CS but optimal clinical response with CyC therapy.

**Case description:** A 46 year old female presented with a three week history of exertional breathlessness, costochondritis, weight loss, lethargy and arthralgia in her feet. Initial investigation revealed crackles in her chest with tenderness at costochondral junction. Her CRP was raised to 260, ANCA/ANA/viral screen was negative, pro-BNP was 74, CTPA was normal, blood cultures were negative and echocardiogram did not show any vegetation. Her flow volume loop showed severe airflow obstruction, FVC 2.40 (68% predicted), FEV1/FVC was 36% (45% predicted) with reduced gas transfer. The initial PET-FDG showed tracer activity in all costochondral joints, glottic and supra-glottic regions, and no FDG activity in the eye, auricular or nasal cartilage. She was initiated with 60 mg of oral prednisolone alongside PPI and calcium/vitamin D supplement. Unfortunately, she remained symptomatic following initial induction therapy with oral CS with persistent laryngeal stridor, breathlessness and high CRP. She was then commenced on 500mg of IVMP for three days followed by CyC therapy (15mg/KG- 6 cycles). Following this, her CRP improved to 30 but she remained breathless and developed a hoarse voice and nasal deformity. A repeat CT showed ongoing disease activity, thickened trachea and airway narrowing. She was readmitted four weeks later for 2nd cycle of IVMP and referred for consideration of tracheal stenting. Following the 2nd cycle of IVMP and completion of six cycles of CYC therapy there was improvement of her clinical symptoms with normalization of CRP. A satisfactory clinical outcome was achieved and a repeat PET-FDG showed significant improvement, but persistent diffuse tracheal thickening and narrowing. Presently, she is on azathioprine 50mg/daily with tapering dose of oral steroid.

**Discussion:** Relapsing polychondritis is an uncommon systemic disease characterized by recurrent episodes of inflammation and destruction of systemic cartilaginous tissues. It has been reported that approximately 50% of patients with relapsing polychondritis have suffered tracheal stenosis and pneumonia, which defines their prognosis (i.e., 5-year and 10-year survival rate are 74% and 55%, respectively. The airway stenosis in relapsing polychondritis is generally caused by intraluminal fibrosis, which, in turn, is caused by inflammation of the tracheobronchial cartilage. This often requires stent therapy. There is limited evidence to guide optimum therapy. Current approaches are based on case reports, case series and expert opinion.

**Key Learning Points:** Relapsing polychondritis is an uncommon condition of cartilage destruction, which is life threatening when it affects the trachea or aortic ring. Tracheal disease is the presenting feature in 10-15% of cases. Our patient failed to respond adequately to oral steroid alone, and needed combination IV CS and CyC therapy to control inflammation. Our case studies also suggest CYC is an effective therapy for severe cases of RP refractory to CS alone.

**Disclosure: M. Solaiman:** None. **O. Ogunbambi:** None.

